# Transcutaneous electrical nerve stimulation reduces exercise-induced perceived pain and improves endurance exercise performance

**DOI:** 10.1007/s00421-016-3532-6

**Published:** 2017-02-03

**Authors:** Ali H. Y. Astokorki, Alexis R. Mauger

**Affiliations:** Endurance Research Group, School of Sport and Exercise Sciences, University of Kent, Chatham Maritime, Chatham, ME4 4AG UK

**Keywords:** Exercise-induced pain, Time to exhaustion, Time trial, Exercise, Gate control theory

## Abstract

**Purpose:**

Muscle pain is a natural consequence of intense and prolonged exercise and has been suggested to be a limiter of performance. Transcutaneous electrical nerve stimulation (TENS) and interferential current (IFC) have been shown to reduce both chronic and acute pain in a variety of conditions. This study sought to ascertain whether TENS and IFC could reduce exercise-induced pain (EIP) and whether this would affect exercise performance. It was hypothesised that TENS and IFC would reduce EIP and result in an improved exercise performance.

**Methods:**

In two parts, 18 (Part I) and 22 (Part II) healthy male and female participants completed an isometric contraction of the dominant bicep until exhaustion (Part I) and a 16.1 km cycling time trial as quickly as they could (Part II) whilst receiving TENS, IFC, and a SHAM placebo in a repeated measures, randomised cross-over, and placebo-controlled design. Perceived EIP was recorded in both tasks using a validated subjective scale.

**Results:**

In Part I, TENS significantly reduced perceived EIP (mean reduction of 12%) during the isometric contraction (*P* = 0.006) and significantly improved participants’ time to exhaustion by a mean of 38% (*P* = 0.02). In Part II, TENS significantly improved (*P* = 0.003) participants’ time trial completion time (~2% improvement) through an increased mean power output.

**Conclusion:**

These findings demonstrate that TENS can attenuate perceived EIP in a healthy population and that doing so significantly improves endurance performance in both submaximal isometric single limb exercise and whole-body dynamic exercise.

## Introduction

Exercise-induced muscle pain (EIP) arises from an accumulation of endogenous algesic substances and an increase in intramuscular pressure (Cook et al. 1997). These endogenous algesics are released from cells when homoeostasis is disturbed as a consequence of intense exercise (Mauger et al. 2010). Therefore, EIP is closely bound to both the intensity and duration of the exercise task (Cook et al. 1997). It is suggested that the perceived pain arising from exercise may play a combined role in the regulation of the level of exercise intensity and preservation of a metabolic reserve by the central nervous system (Mauger 2014). However, the means by which this may occur is complex, and likely involves both physiological and psychological mechanisms. Indeed, increased activity of afferent fibres, which are stimulated by muscle nociceptors, can reduce maximal voluntary contraction of a muscle (Graven-Nielsen et al. 2002; Kennedy et al. 2013) and could reduce exercise performance through a reduction in voluntary activation (Kennedy et al. 2013). Thus, muscle pain may increase afferent neuron inhibition and obstruct or alter the ability of the brain to recruit muscle to produce force (Graven-Nielsen et al. 2002), which would ultimately contribute to fatigue and a decreased task performance. In addition to this, perceived pain provides a powerful psychological stimulus to disengage with the behaviour or action that is causing it. As EIP increases linearly with exercise intensity, it is suggested that intolerable EIP may influence decisions to reduce work rate (to reduce pain), or disengage with the exercise task (Kress and Stratler 2007; Mauger 2014). In both these instances, an impeded endurance performance will be the result. In support of this notion, individuals who are willing to tolerate more pain demonstrate superior endurance performances than those who are not (Astokorki and Mauger 2016), and reducing pain during exercise can result in an improved endurance performance (Mauger et al. 2010; Foster et al. 2014).

Transcutaneous electrical nerve stimulation (TENS) and interferential current (IFC) have been shown to elicit analgesic effects in a variety of conditions (Marchand et al. 1993; Robinson 1996; Salisbury and Johnson 1997). The neurophysiological basis of muscle pain relief from TENS is believed to derive directly from the gate control theory of pain. Here, it is suggested that non-‎nociceptive afferent fibres activate interneurons at the spinal cord level which inhibit the activity of ‎nociceptive projection neurons, thus blunting nociceptive input from the peripheral tissues. Accordingly, TENS is proposed to selectively activate Aβ large-diameter afferent fibres by high-frequency stimulation, inhibiting constant transmission of nociceptive neurons by generating an afferent barrage of nerve impulses within the spinal cord (Melzack and Wall 1967). It is also suggested that the application of TENS burst mode can lead to the release of endogenous opioids, and serotonin, and a subsequent decrease in muscle pain (Sabino et al. 2008). IFC utilises a medium frequency alternating current with a various beat frequency (Gomes et al. 2014), which is believed to reduce pain transmission through gate control mechanisms, release of endorphins, and increased circulation of opioids (Melzack and Wall 1967).

Muscle stimulation using therapeutic current has previously been used in combination with exercise to achieve a variety of effects, including facilitated recovery and relief from delayed onset muscle soreness (Heyman et al. 2009; Vanderthommen et al. 2012). Given that EIP may be a factor affecting exercise capacity and performance and that therapeutic muscle stimulation has shown promise in the treatment of muscle pain (Tourville et al. 2006), there may be scope to use this technique to reduce naturally occurring muscle pain during exercise.

No studies have considered the effectiveness of TENS and IFC on EIP during fatiguing exercise. Therefore, the aim of this study was to investigate whether TENS or IFC elicit an analgesic effect during exercise, and whether this would improve endurance exercise performance. It was hypothesised that TENS and IFC would reduce perceived EIP, assessed using the Cook Scale (Cook et al. 1997), and improve participants’ endurance performance.

## Methods

### Participants and experimental design

Prior to participation in either parts (Part I and Part II) of this study, an information sheet was given to participants, which included an inclusion/exclusion criteria checklist. Participants were excluded from the study if they had history of cardiovascular disorders (e.g., angina, heart attack, high blood pressure, etc), chronic medications that affect the central nervous system, current pregnancy, bleeding disorders (e.g., haemophilia), deep vein thrombosis, impaired sensation, acute/chronic infection (e.g., tuberculosis), malignancy, recently radiated tissue, skin diseases or severely damaged skin, types I or II diabetes, were using a cochlear implant hearing device or pacemakers, or any other condition that may be a danger to their participation (e.g., muscle injury). Following satisfactory completion of the inclusion/exclusion criteria checklist, all participants provided written informed consent and the research was approved by the University Ethics Committee (Reference Number: Prop 69_2014_2015 and Prop 146–2014_2015). Prior to all experimental occasions, participants were asked to refrain from the ingestion of alcohol 48 h before the laboratory visits, and asked to refrain from any vigorous exercise (24 h prior), caffeine (8 h prior), and analgesics (6 h prior) prior to any test occasion.

This study comprised of two parts (Part I and Part II). One participant completed both Part I and Part II of this study. The purpose of Part I was to demonstrate a proof of principle that TENS and IFC are able to attenuate EIP. Part II was subsequently conducted to ascertain whether the reduction in EIP from TENS and IFC would elicit an improvement in endurance exercise performance. The two separates parts of this study were necessary, because where interventions to reduce pain during self-paced exercise have been previously used (e.g., Mauger et al. 2010), participants usually appear to moderate their work rate to maintain the same linear progression of EIP. Thus, in the current study, Part I provided an exercise task that could demonstrate a reduction of perceived EIP as a result of the intervention, which could then be directly attributed to any potential performance effect during whole body exercise in Part II. As both parts of this study followed a repeated measures design, all participants performed all conditions in a randomised, crossover, and placebo-controlled design‎.

## Part I

### Participants and experimental design

Eighteen recreationally active male (*n* = 11) and female (*n* = 7) participants were recruited for Part I of this study. Sample size was estimated from power calculations using a commercially available software package (GPower) and with mean and SD data from similar exercise and pain studies (Mauger et al. 2010; Foster et al. 2014). The participants’ mean age, height, body mass, and peak biceps flexion force were 25 ± 6 years, 176 ± 11 cm, 73.5 ± 16.6 kg, 200 ± 65 N, respectively. Participants attended the laboratory on four occasions (a familiarisation visit and three experimental conditions) at the same time of day (±2 h) to help control for potential circadian influence on psychological variables pertinent to this study (Gobbo and Falciati 2014). The first laboratory visit provided a familiarisation (FAM) of all experimental procedures to reduce learning effects, and this was followed by three further visits that involved a TENS intervention, an IFC intervention, and a placebo-controlled condition (SHAM). TENS, IFC, and SHAM were completed in a single-blind, randomised, and placebo-controlled design.

### Familiarisation (FAM)

Participants were initially tested for sensory discrimination using a sharp and blunt patella hammer and a skin integrity test to ensure normal skin sensation. Following this, participants underwent a full familiarisation of TENS and IFC, which also provided confirmation for subsequent experimental visits that the applied current intensity elicited a tingling sensation without muscle contraction and/or muscle pain (i.e., non-painful paraesthesia). During stimulation, and after testing, participants were monitored for signs of skin irritation, nausea, swelling, and pain. Following this, participants were introduced to the standard instructions for the numeric perceived pain rating scale (Cook et al. 1997) and rating of perceived exertion (RPE) using the Borg (6–20) (Borg 1998) scale. Participants were instructed to report RPE solely as effort to drive the limb (Pageaux et al. 2015) (i.e., independent of pain and discomfort) and that pain should be anchored to exercise-induced pain (i.e., numeric values given relative to their experience of muscle pain). After participants confirmed their understanding of the pain and RPE scales, participants were familiarised with performing a maximal voluntary contraction (MVC) of their dominant arm and the time to exhaustion (TTE) test.

### Application of TENS, IFC, and SHAM

Prior to electrode placement and to reduce electrical resistance, the skin over the biceps of the dominant arm was cleaned thoroughly. Following this, bipolar surface electrodes were attached to the belly of the biceps of the dominant arm 2.5 cm apart. This location was marked, so that the placement would remain consistent between visits. Using a Vectra Genisys multi-waveform stimulator (Chattanooga Group, Hixon, TN, USA), the parameters of biphasic IFC pulses were delivered in a continuous mode with a pulse frequency of 100 Hz. For the biphasic TENS pulses, a continuous pattern of stimulation was used, with a pulse width of 300 μs and a frequency of 100 Hz. A bipolar IFC setup was used in the current study to maintain blinding of conditions. Both bipolar and quadripolar IFC have been shown to be equally successful when used to manage pain conditions (Salisbury and Johnson 1995). The current intensity for the TENS and IFC conditions was selected, so that participants reached a strong but appropriate intensity without causing any noticeable muscle contraction. Stimulation was applied for 5 min prior to, and throughout the TTE test and during the pre- and post MVC. A SHAM stimulation was used as a placebo-controlled condition. During the SHAM condition, electrodes were placed in the same locations as the IFC and TENS conditions, but participants received no current and were told “This type of stimulation is supposed to reduce pain by using a subthreshold stimulus that you will not able to perceive”. This explanation was strengthened via a visual display of the electrical current on an oscilloscope.

### Maximal voluntary contraction (MVC)

To ensure that maximal effort was given during the TTE and that muscle fatigue occurred as a result, an MVC was completed prior to, and immediately after completion of the TTE test. The pre-MVC test also served to set the target force for the TTE test on that experimental visit. Following a warm-up, participants performed three unilateral (dominant arm) maximal voluntary contractions (MVC) of the elbow flexors against a load cell (Globus Ergo Meter, Globus, Codogne, Italy), which were separated by 3 min rest. To do this, participants were in a seated position with the upper arm resting on a bench and the elbow angle at 90° and the wrist angle at 180°. Arm angles for the MVC and TTE tests were measured using a goniometer, and participant body position was maintained both during the tests and between visits by standardising the sitting position for each participant. Each MVC test was performed for 5 s with a rapid increase in force over 1 s, a sustained maximum for 3 s, and a gradual release over the final second. Maximal force was recorded for each MVC. Participants were strongly encouraged to perform maximally throughout each contraction. The maximum of the three values was used to establish the 20% MVC for the time to exhaustion task (TTE) performed in that visit. On completion of the TTE task, participants performed a final, single MVC.

### Time to exhaustion (TTE) test

A rest period of 10 min was provided after the pre-MVCs. Following this, participants undertook the TTE in the same standardised seated position described for the MVC tests. The TTE task required the participant to maintain a 20% isometric MVC of the biceps until the force dropped for 2 s, or when the participant withdrew from the task. During the TTE task, participants were asked to rate their perceived pain and RPE every 30 s. The experimenter provided no encouragement and sat out of sight from the participants.

In visits 2–4, participants completed a pre-MVC, followed by the TTE test and a post MVC immediately after, with either TENS, IFC, or SHAM stimulation being applied during the TTE.

## Part II

### Participants and experimental design

Twenty-two participants (male *n* = 14, female *n* = 8), trained in cycling or triathlon and exercising regularly (>3 h per week), were recruited for this study. Sample size was estimated with a commercially available software package (GPower) and with mean and SD data from similar exercise and pain studies (Mauger et al. 2010; Foster et al. 2014). The participants’ mean age, height and body mass, VO_2max_, and peak power output were 33 ± 8 years, 173 ± 7 cm, 71.8 ± 13.3 kg, 53 ± 7 ml/min/kg, and 286 ± 75 (W), respectively. Participants attended the laboratory on four occasions at the same time of day (±2 h) to complete a full familiarisation (FAM) of all experimental procedures, two experimental visits (TENS and IFC), and a placebo-controlled condition (SHAM). TENS, IFC, and SHAM were completed in a single-blind, randomised, and counter-balanced design.

### Familiarisation

On the first visit to the laboratory, participants underwent the same screening, stimulation, and perceptual scale familiarisations described in Part I. Following this, participants completed a graded exercise test (GXT) to exhaustion. Following a 30 min recovery period, participants completed a 10 mile (16.1 km) cycling time trial (TT) as fast as they could to provide a familiarisation of this task for the subsequent three experimental visits.

### Graded exercise test (GXT)

Following a standardised 10-min warm-up at a self-selected intensity on the cycle ergometer (Velotron, Racermate, Seattle, WA), participants completed an incremental step protocol to exhaustion. Power output (PO) started at 100 W with increases of 30 W. min^−2^, and participants maintained a self-selected cadence until volitional exhaustion or when they could no longer maintain the required cadence. During the test, gas exchange (Cortex Metalyser 3B, Cortex GmbH, Lepzig, Germany) and heart rate (HR) (Polar Electro, N2965, Finland) were recorded continuously, with RPE and perceived pain recorded at the end of each stage. Throughout the test, verbal encouragement was given by the researcher. On completion of the test, participants received a 30 min rest period during which they were familiarised with, and completed, a mood questionnaire [Brunel Mood Scale (BRUMS)] (Terry et al. 2003).

### Ten mile time trial (TT)

To provide a measure of endurance performance, participants were instructed to complete a 10-mile (16.1-km) cycling time trial (TT) on the cycle ergometer (Velotron, Racermate, Seattle, WA) in the fastest possible time. Participants could change gear and cadence to vary their PO, and they could see the distance they had completed but were given no information on performance or physiological parameters (e.g., PO, HR, and time elapsed). Participants were asked to report RPE and perceived pain every km completed. A fingertip sample of blood was acquired every 4 km for analysis of blood lactate concentration (B[La]).

### TENS, IFC, and SHAM stimulation

TENS, IFC, and SHAM were applied in visits 2–4. The same stimulation parameters and procedures described in Part I were also used for Part II. However, stimulation was applied to the belly of the vastus lateralis of both thighs, rather than the biceps. To assess potential differences in mood at baseline between conditions, and following the TT, a BRUMS was completed on entry to the laboratory and following completion of the TT.

### Data and statistical analysis

Prior to statistical analysis, the standard assumptions were checked for each statistical test, and none of these were violated. Time to exhaustion (TTE) and TT completion time were analysed using repeated measures ANOVA and Bonferroni Pairwise Comparisons. Main effect and interaction effects for EIP, RPE, B[La], PO, and HR were assessed using three-way ANOVA with repeated measures, with follow-up paired samples *t* tests used to detect differences between conditions when an interaction effect had been observed. All statistical analysis was performed using the statistical package SPSS version 22 for Windows. Descriptive data are reported as means ± SD. Statistical significance was accepted when *P* < 0.05.

## Results

### Part I

#### Time to Exhaustion (TTE)

**Fig. 1:**
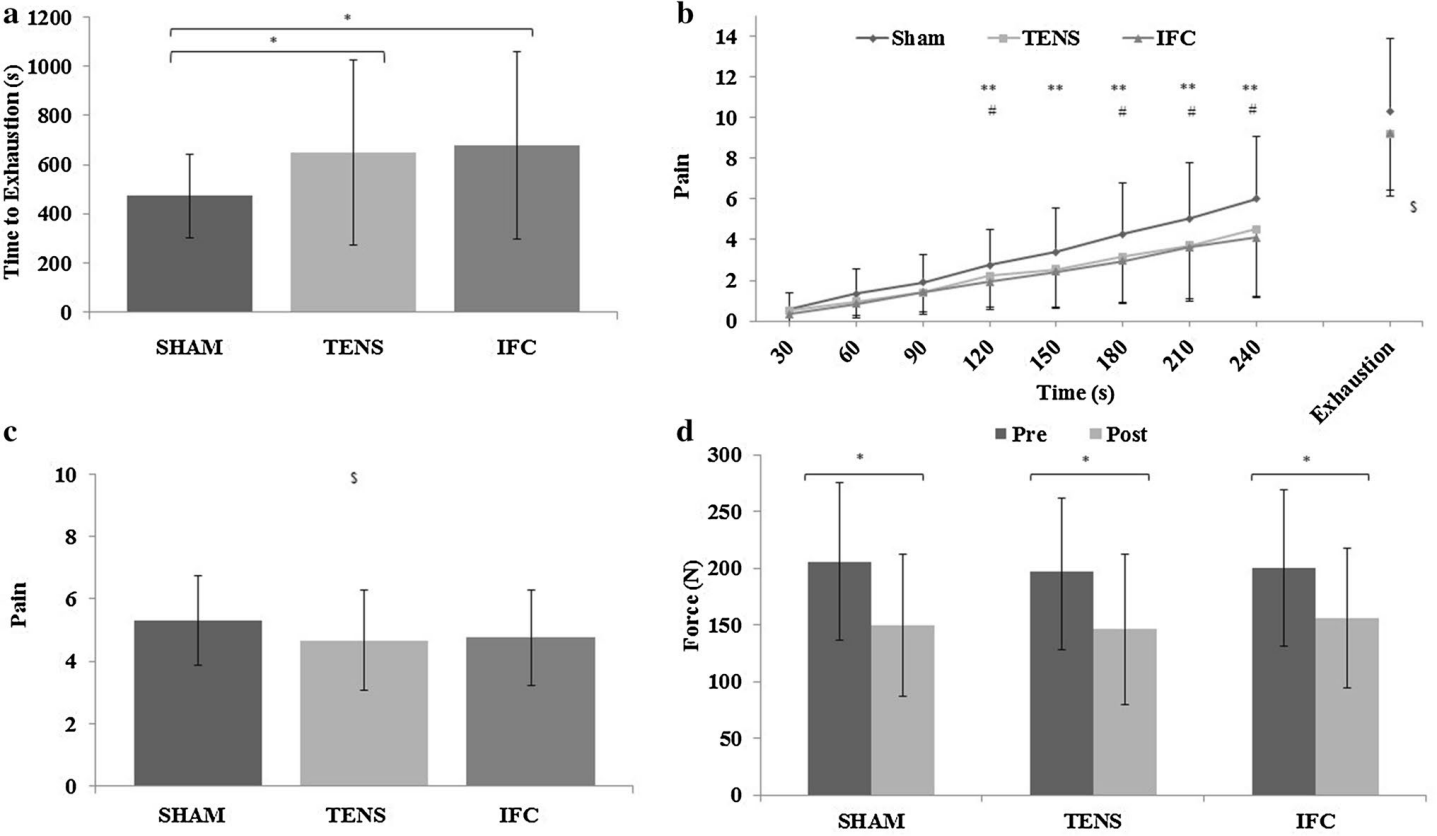
Performance and perceptual differences between conditions in Part I. **a** TTE differences between conditions. **b** Exercise-induced pain intensity over time between conditions during the TTE test. **c** Differences in mean exercise-induced pain intensity between conditions over the TTE test. **d** Maximal voluntary contraction values for pre- and post-TTE tests between conditions. *Significant difference (*P* < 0.05). **Significant difference between IFC and SHAM (*P* < 0.05). ^#^Significant difference between TENS and SHAM (*P* < 0.05). ^$^Main effect for condition (*P* < 0.05)

ANOVA revealed a significant difference in the time to exhaustion between conditions (*F*_(2, 34)_ = 6.763, *P* = 0.003), as shown in Fig. 1a. Pairwise comparisons revealed a significantly different TTE time between TENS (10 min 49 s ± 6 min 16 s) and SHAM conditions (7 min 52 s ± 2 min 51 s) (*P* = 0.31) and between IFC (11 min 17 s ± 6 min 23 s) and SHAM conditions (*P* = 0.02). No significant difference between TENS and IFC conditions was observed (*P* > 0.05).

#### Exercise-induced pain (EIP)

A 3 × 8 ANOVA revealed a significant main effect of condition for perceived exercise-induced pain (*F*_(1.24, 19.13)_ = 8.39, *P* = 0.006). There was also a significant main effect for time (*P* < 0.001). There was also a significant interaction effect for exercise-induced pain over time between conditions during the TTE test (*F*_(3.73, 63.4)_ = 4.95, *P* = 0.002), as shown in Fig. 1b. Follow-up paired-sample *t* tests showed a significantly different pain perception between TENS and SHAM conditions at 120 s (*t*_(17)_ = 2.482, *P* = 0.024), 180 s (*t*_(17)_ = 2.319, *P* = 0.033), 210 s (*t*_(17)_ = 3.402, *P* = 0.003), and 240 s *t*_(17)_ = 3.589, *P* = 0.002. Significant differences were also shown between IFC and SHAM conditions at 120 s (*t*_(17)_ = 2.482, *P* = 0.024), 150 s (*t*_(17)_ = 2.388, *P* = 0.029), 180 s (*t*_(17)_ = 2.997, *P* = 0.008), 210 s (*t*_(17)_ = 3.298, *P* = 0.004), and 240 s (*t*_(17)_ = 2.858, *P* = 0.011). No differences were found at any timepoint between TENS and IFC conditions (*P* > 0.05).

#### Rating of perceived exertion (RPE)

There was no significant main effect of condition (*F*_(2, 34)_ = 2.71, *P* = 0.081) for RPE. There was a main effect for time (*P* < 0.001). No interaction effects for RPE during the TTE were observed (*F*_(4.08, 69.39)_ = 1.82, *P* = 0.134).

#### Maximal voluntary contraction (MVC)

No significant differences between conditions were found for the pre-MVC (*F*_(1.4, 23.4)_ = 1.758, *P* = 0.188) or the post-MVC (*F*_(2, 34)_ = 1.499, *P* = 0.238). MVC was significantly reduced following the TTE in the SHAM (*t*_(17)_ = 9.069, *P* < 0.001), TENS (*t*_(17)_ = 7.037, *P* < 0.001) and IFC conditions (*t*_(17)_ = 8.558, *P* < 0.001), as shown in Fig. 1d, suggesting that significant fatigue and performance decrement had occurred in all conditions following the TTE task.

### Part II

#### Time trial (TT) completion time

ANOVA revealed a significant difference in completion time between conditions (*F*_(2, 42)_ = 6.597, *P* = 0.003). Pairwise comparisons revealed that participants performed a significantly faster TT (*P* = 0.001) in the TENS condition (29 min 6 s ± 3 min 20 s) compared to the SHAM (29 min 39 s ± 3 min 34 s) condition. There were no significant differences (*P* = 0.872) between the IFC condition (29 min 28 s ± 3 min 34 s) and the SHAM, or the TENS and IFC conditions (*P* = 0.116).

#### Power output (PO)

**Fig. 2:**
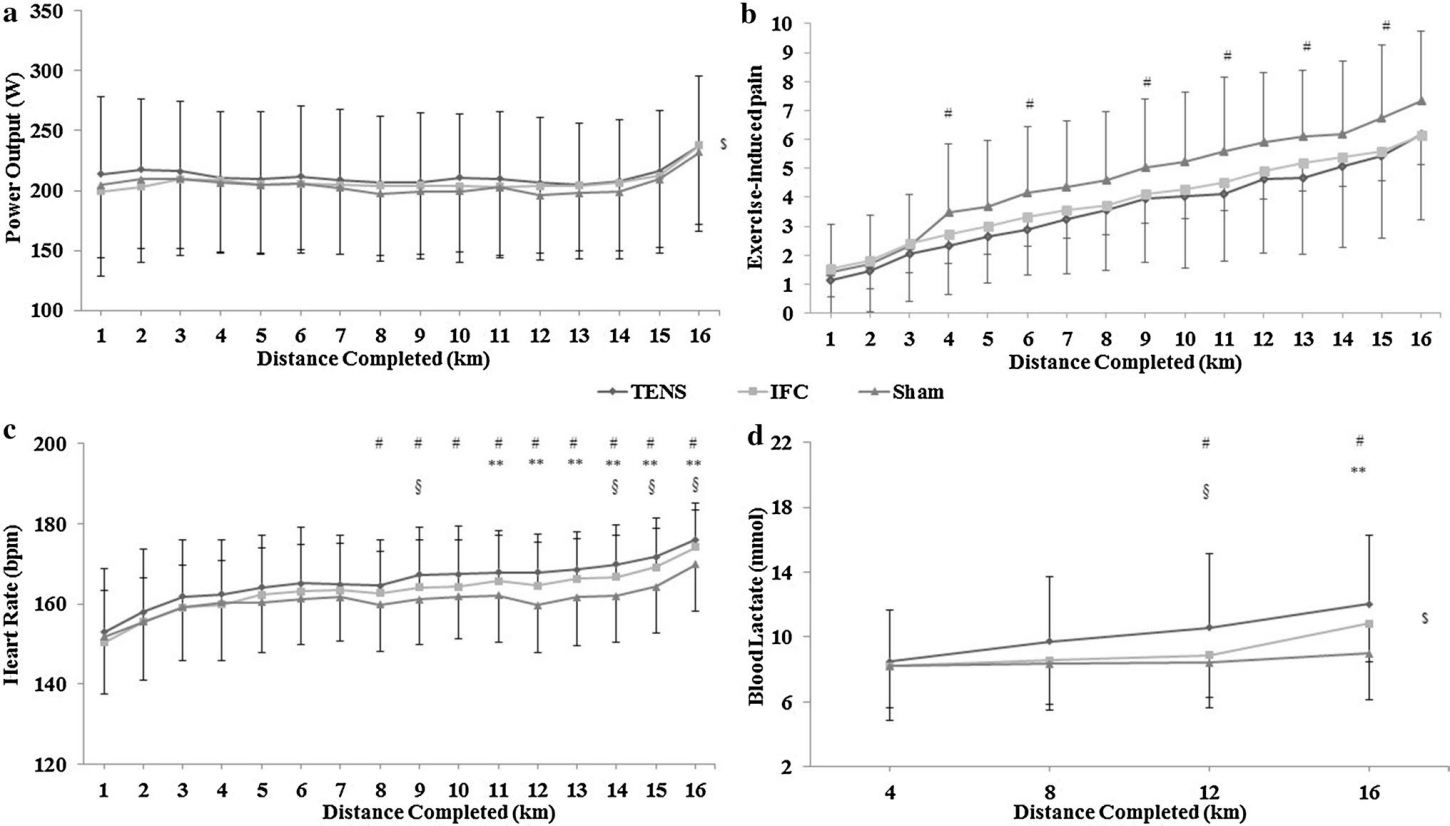
Performance, physiological, and perceptual differences between conditions over time during the time trial in Part II. **a** Power output differences between conditions over time. **b** Exercise-induced pain intensity over time between conditions. **c** Heart rate values between conditions. **d** Blood lactate concentration between conditions. ^#^Significant difference between TENS and SHAM (*P* < 0.05). **Significant difference between IFC and SHAM (*P* < 0.05). ^§^Significant difference between TENS and IFC (*P* < 0.05). ^$^Main effect for condition (*P* < 0.05)

ANOVA revealed a significant main effect of condition for power output (*F*_(2, 38)_ = 3.48, *P* = 0.041). There was also a main effect for distance completed (*P* < 0.001), but no interaction effect (*F*_(30, 570)_ = 0.92, *P* = 0.587), as shown in Fig. 2a.

#### Rating perceived exertion (RPE)

No significant main effects for condition were observed (*P* > 0.05). There was a main effect for distance completed (*P* < 0.001), but no significant interaction effect was found (*P* > 0.05).

#### Exercise-induced pain (EIP)

There was no main effect of condition for EIP (*F*_(1.41, 29.62)_ = 3.60, *P* = 0.054). There was a significant main effect for distance completed (*P* < 0.001) and a significant interaction effect (*F*_(30, 630)_ = 2.04, *P* = 0.001). Follow-up paired *t* tests revealed that participants perceived significantly less EIP in the TENS condition compared to the SHAM at the 4th, 6th, 9th, 11th, 12th, 13th, and 15th km (*P* < 0.05), as shown in Fig. 2b.

#### Heart rate (HR)

ANOVA revealed a significant difference in the mean HR between conditions during the TT (*F*_(1.38, 29.06)_ = 4.016, *P* = 0.042). There was a significant main effect for distance completed (*P* < 0.05), and a significant interaction effect was observed (*F*_(1.3, 27.8)_ = 3.171, *P* = 0.008). Follow-up paired-sample *t* tests showed a significant difference in HR between TENS and SHAM conditions between 8th and 16th km (*P* < 0.05). In addition, significant differences in HR between IFC and SHAM conditions were observed between 11th and 16th km (*P* < 0.05). There were also significant differences in HR between TENS and IFC conditions during 9th, 14th, 15th, and 16th km (*P* < 0.05). Differences in HR between conditions are shown in Fig. 2c.

#### Blood lactate B[La]

ANOVA revealed a significant main effect of condition (*F*_(1.49, 31.37)_ = 7.54, *P* = 0.004), a main effect for distance completed (*P* < 0.05) and a significant interaction effect *F*_(3.68, 77.63)_ = 3.51, *P* = 0.013. Follow-up paired-sample *t* tests showed a significantly different B[La] between TENS and SHAM conditions at 12th km (*t*_(21)_ = −2.850, *P* = 0.01) and 16th km (*t*_(21)_ = −4.370, *P* < 0.001). There was also a difference in B[La] between IFC and SHAM conditions at 16th km (*t*_(21)_ = −3.632, *P* = 0.002) and a significant difference in B[La] between TENS and IFC conditions at 12th km (*t*_(21)_ = 2.496, *P* = 0.021), as shown in Fig. 2d.

#### BRUMS

No differences in mood states were found between conditions pre- or post-TT. Paired-sample *t* tests showed a significant difference for vigour from pre- to post-TT during the TENS condition (*t*_(21)_ = −2.114, *P* = 0.047). No other differences in pre–post-mood states were observed.

## Discussion

This study investigated whether TENS and IFC can moderate EIP, and whether this would lead to an improvement in endurance performance. The primary finding was that both TENS and IFC were able to significantly reduce EIP during single limb exercise, but only TENS was capable improving whole-body endurance performance. This is the first study utilising a randomised, crossover, and placebo-controlled design, which shows an ergogenic effect for TENS. This study also provides support for the notion that EIP is a limiter of endurance performance in both single limb and whole-body exhaustive exercise. Although pain tolerance has long been linked to athletic potential (Scott and Gijsbers 1981), it is only relatively recently that a growing body of empirical evidence has provided strong support for this notion. EIP may exacerbate fatigue by reducing voluntary activation of the muscle (Kennedy et al. 2013) or by contributing to a host of unpleasant sensations (Kress and Stratler 2007) that either leads to a decision to reduce work rate or disengage with the task (Mauger 2014). Whilst the current study cannot identify whether psychological or physiological determinants led to the apparent ergogenic effect of therapeutic muscle stimulation, it does provide further evidence that analgesic interventions during exercise can improve endurance performance.

In both the parts of the study, EIP increased as a function of time and reached its most intense at the end of the exercise, where near maximal values were observed. To moderate this pain, without changing the metabolic environment at the muscle, TENS and IFC were used to inhibit the transmission of the nociceptive signal at the spinal level. The TENS intervention appeared to reduce perceived pain, which resulted in a longer time to exhaustion of the sustained isometric contraction and a faster TT time. The analgesic mechanism of TENS and IFC is suggested to be underpinned by the gate-control theory of pain (Sluka and Walsh 2003). Indeed, when TENS and IFC are applied to produce a strong comfortable and non-painful paraesthesia, large diameter afferents (A-beta fibres) are selectively activated (Sluka and Walsh 2003). The activation of these large diameter low threshold mechano-receptive nerve fibres could inhibit the nociceptive transmission from small diameter higher threshold nociceptive (A-delta and C) fibres through pre- and post-synaptic inhibition in the dorsal horn of the spinal cord (Melzack and Wall 1967). This would reduce the number of nociceptive signals reaching the higher brain centres and consequently reduce the perceived pain for a given stimulus at the nociceptor. A reduction in the afferent barrage from Type III and IV fibres could also offset the reduction in voluntary activation that is observed during painful exercise (Kennedy et al. 2013), which would likely allow for an improved exercise performance. Analgesia through TENS and IFC may also be explained by the release of endogenous opioids (Sabino et al. 2008). Whilst evidence for this mechanism is stronger for low-frequency TENS (Sjölund and Eriksson 1979), more recent studies on animal models also suggest that analgesia by high-frequency TENS is reduced by systemic naloxone in high enough dose to block μ, δ, and κ opioid receptors (Han et al. 1991), thus supporting a role for endogenous opioids for both high- and low-frequency TENS. The observation that IFC only provided an analgesic advantage in single limb exercise is contrary to what was expected, and difficult to reconcile. The most likely reason is that whilst TENS is suggested to primarily operate according to Gate Control Theory, IFC involves modulation of the transmission of pain through the release of endogenous opioids (Sabino et al. 2008). Ray and Carter (2007) that have previously shown that endogenous opioids do not appear to modulate acute EIP, and so the lack of analgesic effect of IFC could be explained by it primarily operating through this mechanism.

The mean reduction in perceived pain (compared to the SHAM condition) elicited by TENS was approximately 12% during single-limb exercise, with a stronger effect evident later in the exercise (>30% after 180 s—see Fig. 1b). The greater reductions in pain towards the end of exercise are paralleled by the increasingly noxious environment in the muscle and the consequential increased pain (Cook et al. 1997). Therefore, the apparent analgesic effect of the stimulation was most noticeable during a noxious environment that elicited a pain intensity of ~4.3 (‘Somewhat strong pain’) and above on the Cook Scale (Cook et al. 1997). It is important to note that in the familiarisation visits, this scale was anchored specifically according to previously experienced maximum and minimum levels of muscle pain during exercise, rather than a general pain sensation (e.g., dental pain), so as to provide a measure specific to the experiences of EIP. The effectiveness of the analgesia observed in the current study is supported by some studies which have used TENS to reduce pain. Indeed, in a cross-over study investigating neuropathic pain in patients with spinal cord injury, analgesic TENS was shown to elicit a 29–38% improvement on a global relief scale. Furthermore, Bjordal et al. (2003) demonstrated a 26.5% mean reduction in analgesic consumption for post-operative patients following a well-controlled TENS intervention. Salisbury and Johnson (1995) have also shown that TENS increased the cold pain threshold and that IFC decreased cold pain intensity. However, whilst several studies have demonstrated positive analgesic effects of TENS, there are a number of studies that show no such effect (Johnson and Tabasam 1998; Claydon et al. 2008; Gomes et al. 2014). The numerous systematic reviews and meta-analysis (Zeng et al. 2015) on this area suggest that different TENS parameters, patient groups, outcome measures and a lack of placebo controls and randomisation are the reason for the equivocal findings for the effectiveness of TENS. Therefore, in the current study, the use of a placebo-controlled condition, the randomisation of conditions, and the controlled exercise intensity between conditions and participants, presents a robust experimental design that supports the effectiveness of TENS as an analgesic intervention and a role for EIP in endurance performance.

A notable observation in the current study is that endurance performance improved following a reduction in pain, but with no significant change in RPE between conditions. It has been suggested that RPE is the conscious manifestation of afferent information from a host of afferent physiological systems and external cues and that this perception of effort is an important determinant of endurance performance (Tucker 2009). However, there is strong evidence to suggest that the primary generator for perception of effort is the corollary discharge (i.e., an internal signal that arises from centrifugal motor commands) associated with central motor command (McCloskey 1981) and that this is independent from afferent feedback (including pain) from the working muscles and other interoceptors (de Morree et al. 2014). Indeed, feelings of pain and discomfort have often been assessed as part of the perception of effort (Noble and Robertson 1996), although numerous studies have shown that pain and effort can be dissociated (Cook et al. 1997; Pageaux et al. 2015; Angius et al. 2015; Astokorki and Mauger 2016) and are, therefore, distinct entities. By dissociating perception of effort and EIP in the current study, we were able to observe the individual effects of therapeutic muscle stimulation on EIP and RPE and the consequent impact on endurance performance. In-line with our hypothesis, a reduction in EIP paralleled an improvement in TTE and TT performance. This finding supports the view that EIP is a contributing factor to task cessation and self-paced performance (Mauger 2014), but is contrary to the view that endurance performance is primarily determined by perception of effort, as stated by the Psychobiological Model (Marcora 2010). Although this model acknowledges that severe pain (such as that from a muscle strain) would affect motivation (and, therefore, inhibit performance), it suggests that the muscle pain normally experienced during high-intensity aerobic exercise does not limit performance in healthy humans (Marcora 2010). The results of the current study suggest that ‘normal’ EIP experienced during exhaustive exercise does affect performance and that it can be moderated independently of perception of effort. These findings support other studies which demonstrate that an analgesic intervention is able to improve exercise performance in a variety of exercise models (Mauger et al. 2010; Foster et al. 2014) and strengthens the notion (Kress and Statler 2007) that tolerance of EIP is an important prerequisite for endurance performance (Mauger 2013, 2014).

## Conclusion

In conclusion, the findings of this study suggest that TENS can elicit an analgesic effect on EIP during both an exhaustive single limb, submaximal isometric contraction and in whole-body exercise. Reducing pain during this exercise improved endurance performance, without any changes to participants’ perception of effort. Further studies are needed to identify how TENS or IFC elicits an analgesic effect for EIP and the psychophysiological mechanisms underpinning the subsequent improvement in endurance performance.

## Abbreviations

EIP: Exercise-induced pain
ANOVA: Analysis of variance
PO: Power output
HR: Heart rate
B[La]: Blood lactate
MVC: Maximal voluntary contraction
RPE: Ratings of perceived exertion
TENS: Transcutaneous electrical nerve stimulation
IFC: Interferential current
TTE: Time to exhaustion test
Hz: Hertz
GXT: Graded exercise test
TT: Time trial
BRUMS: Brunel Mood Scale
